# Construction Notes

**Published:** 1896-08-15

**Authors:** 


### CONSTRUCTION NOTES.

### Workhouse Infirmary at Newton (Devon).

This building, which will have a frontage of 200 feet, and is of tasteful design, will cost, it is estimated, about £8,000. In the central blocks there will be the surgery, the nurses' duty room, and a separation ward, and behind them there will be two day-rooms, a nurses' sitting-room, and bath-rooms. In the two wings of the building are the four wards for males and females, each ward being 62 feet by 24 feet. The heating is to be by Manchester stoves, and Boyle's ventilators are to be used. Ample means are to be provided for escape in case of fire, and the floors and corridors are to be fireproof. The walls of the corridors and offices are to be lined with white glazed brick. The wards will be provided altogether with 80 beds. At the rear, but disconnected with the main building, and approached by a long corridor, will be the lying-in ward—a one-storeyed building—and here there will be 10 beds. The new block is being erected on an elevated and healthy situation at the rear of the workhouse, and it is expected to be completed for occupation in the winter of 1897.

### Waddon Isolation Hospital.

The Croydon Infectious Diseases Hospital, Waddon, which has been the last two years in course of erection, is now formally opened. A more desirable site than the one occupied could hardly have been selected. The hospital is built on an eminence, the surrounding country consisting of stretches of arable land. Owing to its somewhat exposed position the air is pure and bracing. The much-felt want of an infectious diseases hospital, which has at last been supplied, and the extent of the benefit which this institution is destined to bestow on the borough it is difficult to overestimate. Everything in connection with the building has been arranged after the most approved plans, while all that could possibly be required for the comfort of the patients and nurses and medical staff has been supplied. The various buildings are surrounded by a high wall which entirely separates the various departments from communication with the outside, there being only one method of approaching the hospital from the outer world, and the gardens within the boundaries of the wall are tastefully laid out. The plan of the hospital construction, comprises 15 separate blocks, each one being fitted up in the most elaborate and finished style. The ventilation and heating apparatus are also well up to date. The hospital, in short, is made as attractive as it can possibly be. In the general accommodation block, two brick pavilions are designed to hold 22 beds each, kitchen, nurses' wards, a brick building for paying patients of four rooms, three iron wards to hold 12, 12, and 24 beds respectively, probationary ward and discharging room, patients' laundry, mortuary, emergency, and administration ward; a block at the end of the site with small iron ward is for enteric fever or cholera cases. The cubic amount of air to each bed is 2,100 feet and upwards. Each ward is heated with an open stove, generating a current of warm air; ventilators are above every bed. The bacteriological laboratory will not only be a great boon to the doctors at the hospital but to all the medical profession in Croydon, for there they can make diagnoses, apply tests, &c. Mr. Walker, borough engineer, carried out the heaviest part of the work, while the actual construction of the building has been carried out by Mr. Saunders, of Croydon; Mr. Hazlehurst, having designed the architectural plans.

